# Proteomic alterations in the brain and blood–brain barrier during brain Aβ accumulation in an APP knock-in mouse model of Alzheimer’s disease

**DOI:** 10.1186/s12987-023-00466-9

**Published:** 2023-09-14

**Authors:** Shingo Ito, Ryotaro Yagi, Seiryo Ogata, Takeshi Masuda, Takashi Saito, Takaomi Saido, Sumio Ohtsuki

**Affiliations:** 1 Department of Pharmaceutical Microbiology, Faculty of Life Sciences, Kumamoto University, 5–1 Oe-honmachi, Chuo-ku, Kumamoto, 862–0973 Japan; 2 Department of Pharmaceutical Microbiology, Graduate School of Pharmaceutical Sciences, Kumamoto University, 5–1 Oe-honmachi, Chuo-ku, Kumamoto, 862–0973 Japan; 3 Department of Environmental Medicine and Molecular Toxicology, Tohoku University Graduate School of Medicine, 2–1 Seiryo-machi, Aoba-ku, Sendai, 980–8575 Japan; 4 Present Address: Department of Neurocognitive Science, Institute of Brain Science, Nagoya City University Graduate School of Medical Sciences, 1 Kawasumi, Mizuho-cho, Mizuho-ku, Nagoya, 467–8601 Japan; 5 Laboratory for Proteolytic Neuroscience, RIKEN Center for Brain Science, 2–1 Hirosawa, Wako, Saitama, 351–0198 Japan

**Keywords:** Alzheimer’s disease, Amyloid-β peptide, Blood–brain barrier, Apolipoprotein, Basement membrane, Proteome analysis

## Abstract

**Background:**

Blood–brain barrier (BBB) dysfunction is supposed to be an early event in the development of Alzheimer’s disease (AD). This study aimed to investigate the relationship between BBB alterations and AD progression in terms of amyloid-β peptide (Aβ) accumulation in the brains of humanized amyloid precursor protein knock-in (APP-KI) mice.

**Methods:**

Brain Aβ accumulation was examined using immunohistochemical analysis. Alterations in differentially expressed proteins were determined using sequential window acquisition of all theoretical fragment ion mass spectroscopy (SWATH-MS)-based quantitative proteomics, and Metascape, STRING, Gene Ontology, and KEGG were used for network analyses of altered biological pathways and processes. Statistical significance was determined using the unpaired two-tailed Student’s *t*-test and Welch’s *t*-test for two groups and one-way analysis of variance followed by Tukey’s test for more than two groups. Correlations between two groups were determined using Pearson’s correlation analysis.

**Results:**

Brain Aβ accumulation in APP-KI mice was detectable at 2 months, increased significantly at 5 months, and remained elevated at 12 months of age. The levels of differentially expressed proteins in isolated brain capillaries were higher in younger mice, whereas those in the brain were higher in older mice. Network analyses indicated changes in basement membrane-associated and ribosomal proteins in the brain capillaries. There were no significant changes in key proteins involved in drug or Aβ transport at the BBB. In contrast, solute carrier transporter levels in astrocytes, microglia, and neurons were altered in the brain of older mice. Moreover, the levels of the lipid transporters Apoe and Apoj were upregulated in both the brain and isolated brain capillaries after Aβ accumulation.

**Conclusions:**

Our results suggest that changes in the brain occurred after advanced Aβ accumulation, whereas initial Aβ accumulation was sufficient to cause alterations in the BBB. These findings may help elucidate the role of BBB alterations in AD progression and predict the distribution of drugs across the BBB in the brain of patients with AD.

**Supplementary Information:**

The online version contains supplementary material available at 10.1186/s12987-023-00466-9.

## Background

Pathological and imaging studies have demonstrated that amyloid-β peptide (Aβ) accumulation in the brain is the first pathological change in Alzheimer’s disease (AD), the most common cause of dementia [1]. Studies have also provided evidence of cerebrovascular pathology in the AD brain, including decreased vascular density and changes in blood vessel morphology and basement membrane structure [2–5]. Furthermore, epidemiological data have shown that brain microvascular dysfunction correlates with cognitive decline in AD [6], and increasing evidence suggests that breakdown of brain capillaries occurs in the early stages of AD [7–12].

The blood–brain barrier (BBB) is formed by the endothelial cells of brain capillaries and the encircling astrocyte endfeet, pericytes, and basement membrane. The BBB acts as both a robust physical barrier and a dynamic interface that regulates brain homeostasis and protects the central nervous system (CNS) by facilitating selective molecular transport between the systemic circulation and CNS, and both these aspects seem to be affected in AD. Physically, the BBB consists of tight junctions and the basement membrane [13], and proteins in the tight junctions as well as in the microvascular basement membrane are altered in the AD brain [2, 4, 14]. The crucial role of the BBB in regulating the exchange of solutes between the blood and brain depends on specific transport proteins known as ATP-binding cassette (ABC) and solute carrier (SLC) transporters, which contribute to maintaining the appropriate microenvironment for optimal brain function. ABCB1 (also known as P-glycoprotein [P-gp]) is a major BBB efflux drug transporter [15]. Positron emission tomography (PET) studies have demonstrated reduced ABCB1/P-gp function in the AD brain [16–18]. The ABCB1/P-gp level in brain microvessels inversely correlates with Aβ accumulation, and *Abcb1ab* knockout in an AD mouse model increased Aβ accumulation in the brain [19]. Moreover, P-gp protein expression and transport function is reduced by Aβ(1–40) [20]. SLC family 2 member 1 (SLC2A1; also known as glucose transporter protein type 1 [GLUT1]) is a glucose transporter highly expressed in the BBB [21]. *Slc2a1*^+*/−*^ knockout in an AD mouse model resulted in cerebrovascular degeneration, neuropathology, and cognitive dysfunction [17]. These findings suggest that alterations of transport functions in brain capillaries play a role in the progression of AD. However, the timing of BBB alterations during AD progression and the pathological relationships between them remain unclear.

Several transgenic mouse models have been used to express human Aβ from Aβ precursor protein (APP) in the brain. Second-generation *App* knock-in mouse models of AD harboring Swedish, Beyreuther/Iberian, and Arctic mutations (*App*^NL−G−F/NL−G−F^; APP-KI) exhibit increased Aβ(1–42) production, Aβ pathology, neuroinflammation, and age-dependent cognitive impairment without alterations in the expression levels of APP or other fragments [22]. Thus, this APP-KI mouse model provides a better background for investigating the relationships between BBB alterations and AD pathology progression without the artificial phenotypes observed in first-generation APP-overexpressing mice.

We have previously reported that sequential window acquisition of all theoretical fragment ion mass spectroscopy (SWATH-MS)-based quantitative proteomics is useful for investigating protein changes in the BBB and brain [23]. However, standard brain capillary isolation methods require at least five mouse brains to obtain a sufficient amount of brain capillary tissue of adequate purity [21, 23, 24]. To overcome this issue, we developed a more efficient method for isolating brain capillaries from frozen mouse brains, which allowed us to investigate the BBB properties of individual mice using quantitative proteomic analysis [25].

In this study, we investigated the proteomic alterations in the brains and in capillaries isolated from the brains of APP-KI mice at different stages of brain Aβ accumulation. Our results indicated that changes in the brain occurred after advanced Aβ accumulation, whereas initial Aβ accumulation was sufficient to cause alterations in the BBB. These findings highlight the role of BBB alterations in AD progression and drug distribution in the brain.

## Methods

### Animals

*App*^*NL−G−F/NL−G−F*^ (APP-KI) male mice on a C57BL/6J background were used as the AD model [22], and age-matched C57BL/6 J male mice (purchased from Charles River Laboratories; Kanagawa, Japan) were used as wild-type (WT) controls. Mice were fed a normal diet (CE-2, 12% kcal from fat; CLEA Japan, Tokyo, Japan) and maintained under a 12 h light/dark cycle. All animals were bred at the Center for Animal Resources and Development (CARD) of Kumamoto University. All animal experiments were approved by the Institutional Animal Care and Use Committee of Kumamoto University (No. A2019-031, A2021-041).

### Preparation of brain lysates and brain capillary fractions

Brain capillary isolation from a frozen mouse brain was performed as previously described [21]. Briefly, a single frozen mouse brain was homogenized in 1 mL of homogenizing buffer (101 mM NaCl, 4.6 mM KCl, 2.5 mM CaCl_2_, 1.2 mM KH_2_PO_4_, 1.2 mM MgSO_4_, 15 mM HEPES, pH 7.4) containing stainless steel beads (3.2 mm, 1.8 g; TOMY SEIKO, Tokyo, Japan) using a bead homogenizer (Bead Mill 4; Thermo Fisher Scientific, Waltham, MA, USA). Part of the brain homogenate (50 μL) was collected in a separate tube. The rest of the homogenate was transferred to a new 2 mL tube and centrifuged at 1000 × g for 10 min at 4 °C. The supernatant was removed carefully and the pellet was suspended in 1 mL of homogenizing buffer. An equal volume of 32% w/v dextran/homogenizing buffer was added to the 2 mL tube and mixed by inverting, after which the samples were immediately centrifuged at 4500 × g for 15 min at 4 °C. The pellets were suspended in suspension buffer (200 μL) and filtered through a cell strainer (70 μm). Brain capillaries were collected from the filtered samples using glass beads (0.35–0.5 mm; AS ONE; Osaka, Japan). The glass beads were then washed and added to 500 μL of suspension buffer. The sample was mixed by inversion and centrifuged (3300 × g for 5 min at 4 °C); the pellet was resuspended in 100 μL homogenization buffer, and a portion of the isolated brain capillary fraction was used for microscopic analysis. The rest of the isolated brain capillary fraction sample was lysed in hypotonic buffer by sonication. Protein concentrations were measured using a Pierce BCA protein assay kit (Thermo Fisher Scientific).

### Quantitative proteomics

Samples were digested using the phase-transfer surfactant method as previously described [25, 26]. A TripleTOF 5600 mass spectrometer (Sciex, Framingham, MA, USA) coupled with the Dionex Ultimate 3000 RSLCnano System (Dionex, Sunnyvale, CA, USA) was used for SWATH-MS. The peptides were filtered at a false discovery rate of < 1% for identification and quantification. Proteins were identified using ProteinPilot v.4.5 (Sciex) using mass spectroscopy data from information-dependent acquisition and UniProt mouse reference proteome data. DIA-NN v.1.7 [27] was utilized to analyze the peptide peaks from SWATH data using a spectral library constructed using the identification data. The MaxLFQ algorithm was used to calculate the protein expression levels from the precursor peak areas [27]; the concentration of each protein was calculated as the peak area of the protein obtained by summing the peak areas of all the specific tryptic peptides.

### Immunohistochemistry

Mice were anesthetized using isoflurane (FUJIFILM Wako Pure Chemical Corporation, Osaka, Japan) and perfused with phosphate-buffered saline (PBS) followed by 4% paraformaldehyde (PFA) in PBS. The brains were collected, fixed in 4% PFA/PBS overnight at 4 °C, incubated in 30% sucrose in PBS for 24 h at 4 °C, and embedded in optimal cutting temperature compound. Frozen sections (thickness, 20 μm) were prepared using a cryostat (CM3050 S; Leica, Hessen, Germany). Brain sections were then washed with PBS, heated in antigen activation solution (HistoVT One; Nacalai Tesque, Kyoto, Japan) for 20 min at 70 °C, and incubated in blocking solution (PBS containing 0.3% Triton X-100, 0.1% bovine serum albumin, and 2% donkey serum) for 2 h at 15–25 °C. The sections were then incubated with primary antibodies (anti-human Aβ (N) (82E1) antibody [10323; Immuno-Biological Laboratories, Gunma, Japan], anti-apolipoprotein E antibody [68587; Cell Signaling Technology, Danvers, MA, USA], anti-apolipoprotein J antibody [AF2747; R&D Systems, Minneapolis, MN, USA]) overnight at 4 °C, washed with PBS containing 0.1% Tween 20, and incubated with secondary antibodies (goat anti-mouse IgG H&L [ab150117; Abcam, Cambridge, UK] and goat anti-rabbit IgG H&L [ab175695, Abcam] or donkey anti-goat IgG H&L [ab175704, Abcam]) for 2 h at 15–25 °C. The sections were washed with PBS containing 0.1% Tween 20 and mounted with VECTASHIELD mounting medium containing DAPI (Vector Laboratories, CA, USA). Finally, images were acquired using a confocal microscope (FV3000, Olympus, Tokyo, Japan) and processed using Adobe Photoshop CS6 (Adobe, San Jose, CA, USA).

### Statistical analysis

Unless otherwise indicated, numerical data are expressed as mean ± standard deviation (SD) values. Statistical significance of the differences between the means was determined using the unpaired two-tailed Student’s *t*-test and Welch’s *t*-test for two groups and one-way analysis of variance followed by Tukey’s test for more than two groups. Correlations between two groups were determined using Pearson’s correlation analysis. All analyses were performed using GraphPad Prism 9 (GraphPad Software, San Diego, CA, USA) and Microsoft Excel (Microsoft, Redmond, WA, USA). Metascape (https://metascape.org) [28], STRING (https://string-db.org/), and KEGG (https://www.genome.jp/kegg/kegg_ja.html) were used for network analysis.

## Results

### Changes in protein expression in APP-KI mouse brain following Aβ accumulation with age

**Fig. 1:**
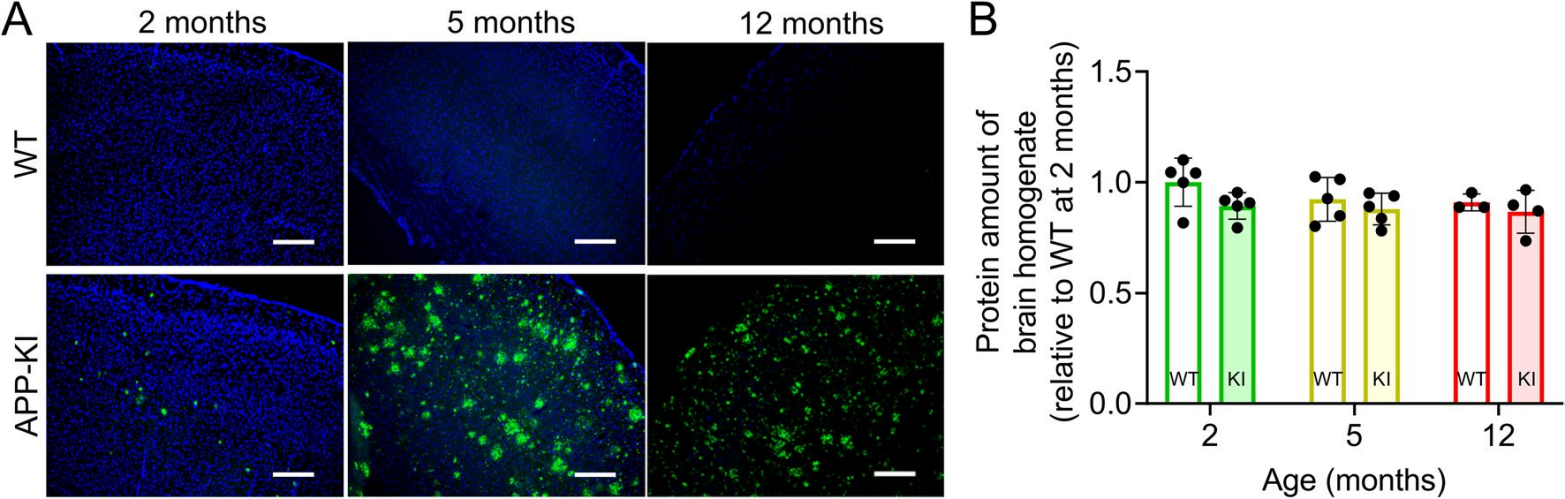
Alterations in brain Aβ accumulation and protein levels in APP-KI mice. **A** Accumulation of Aβ (82E1) in the brains of 2-, 5-, and 12-month-old WT and APP-KI mice. Scale bar: 200 μm. **B** Protein levels in brain homogenates from 2-, 5-, and 12-month-old WT and APP-KI mice. Protein levels were normalized to brain weight and the relative ratio to WT at 2 months. Bars represent mean ± SD values (n = 3–5). Plotted points represent individual values

Immunohistochemical analysis showed that Aβ accumulation in the brain was already visible in 2-month-old APP-KI mice, increased significantly in 5-month-old mice, and remained elevated in 12-month-old mice (Fig. 1A). Thus, 2-, 5-, and 12-month-old humanized APP-KI (*App*^*NL−G−F/NL−G−F*^) mice showed early, intermediate, and advanced Aβ accumulation in the brain, respectively. Protein assays showed that the protein levels in the brain lysates from APP-KI mice were not significantly different from those in lysates from age-matched WT mice (Fig. 1B).

**Fig. 2:**
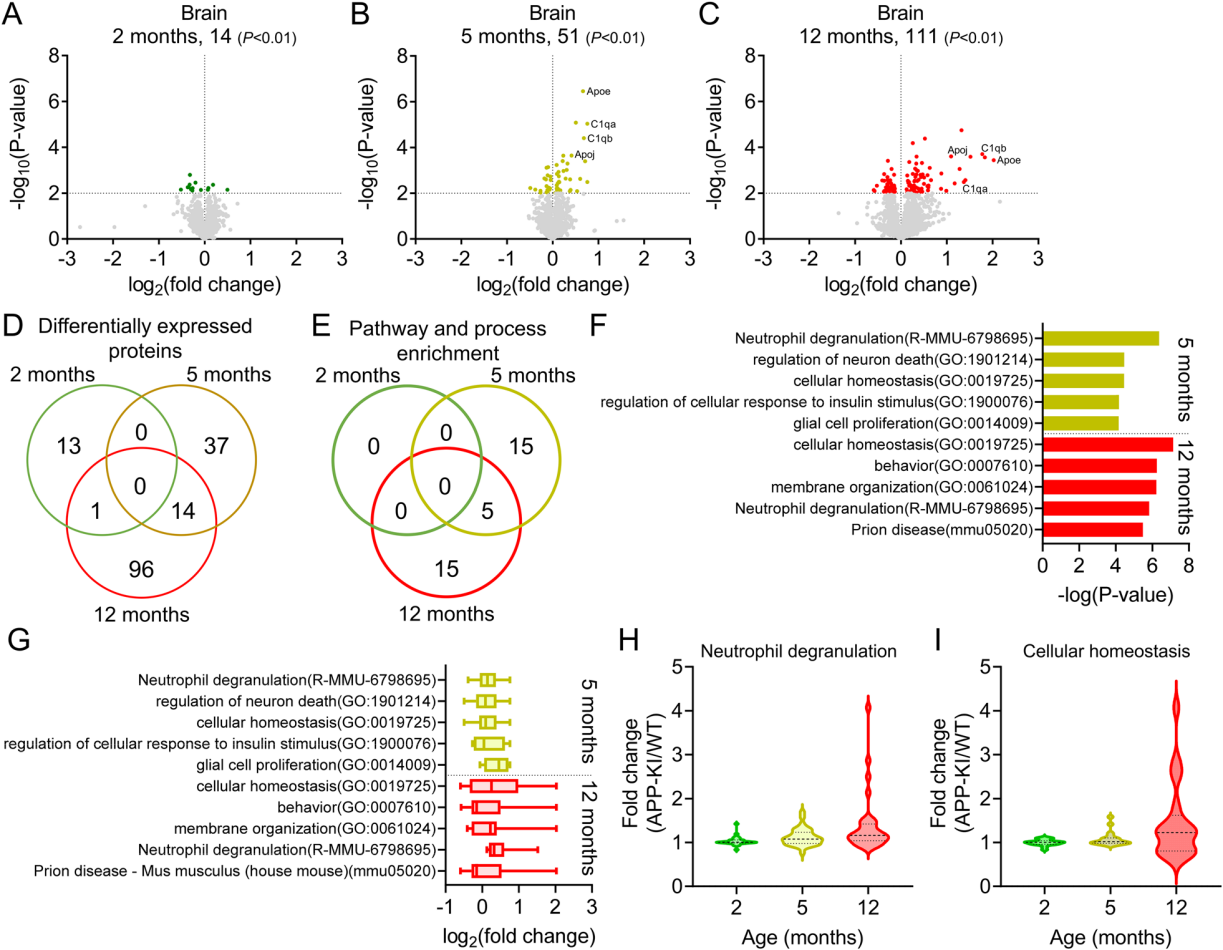
Changes in biological functions based on differentially expressed proteins in the APP-KI mouse brain. **A**–**C** Volcano plots of the identified proteins in the brains of 2-, 5-, and 12-month-old mice. Brain homogenates (from 3–5 biological replicates) were subjected to SWATH-MS-based quantitative proteomic analysis. *P*-values estimated by Welch’s t-test were plotted against fold change (APP-KI/WT) of protein expression for all identified proteins in the brain lysates of age-matched mice. The horizontal line in each graph represents the significance level (*P* < 0.01). The vertical line in each graph represents the fold change (1). **D** Venn diagrams comparing differentially expressed proteins in the brains of 2-, 5-, and 12-month-old APP-KI mice. **E** Venn diagrams comparing the top 20 enriched biological processes identified by Metascape in the brains of 2-, 5-, and 12-month-old APP-KI mice. **F**, **G** Top 5 enriched biological processes (**F**) and changes in the associated protein levels (**G**) in 5- and 12-month-old APP-KI mice. Box-plot elements: center line, median; box limits, upper and lower quartiles; whiskers, minimum and maximum values. (H, I) Age-dependent changes in protein expression associated with neutrophil degranulation (**H**) and cellular homeostasis (**I**) in the brain of APP-KI mice. Violin plot elements: center line, median; upper and lower lines, upper and lower quartiles

Differentially expressed proteins in the brain homogenates of APP-KI and age-matched WT were identified using SWATH-MS-based quantitative proteomic analysis. The median percentage coefficient of variance (%CV) of the intensities and fold changes (APP-KI to WT) of identified proteins in each group was less than 15% (Additional file 2: Fig S1A, B), indicating that the quality of the proteomic data was sufficient for comparative analysis. The number of differentially expressed proteins increased with age in APP-KI mice (*P* < 0.01, Welch’s t-test; Fig. 2A–C), and was approximately 2.2-fold higher in 12-month-old mice than that in 5-month-old APP-KI mice. Fourteen proteins, including Apoe and Apoj, which are major genetic risk factors for AD, and C1qa, and C1qb, which are increased in the AD brain [29], were identified as differentially expressed proteins between 5- and 12-month-old mice (Fig. 2B–D).

To identify alterations in the biological functions of the differentially expressed proteins, pathway and process enrichment analyses were performed using Metascape. No such biological pathways or processes were identified in the brains of the 2-month-old APP-KI mice (Fig. 2E). In contrast, several altered biological pathways and processes were identified in 5- and 12-month-old APP-KI mice (Fig. 2E). The top five of these indicated that the proteins were enriched in biological pathways and processes related to “neutrophil degranulation (R-MMU-6798695)” and “cellular homeostasis (GO:0019725)”. Proteins were also enriched in biological pathways and processes related to “regulation of neuron death (GO:1901214)”, “regulation of cellular response to insulin stimulus (GO:1900076)”, and “glial cell proliferation (GO:0014009)” in 5-month-old APP-KI mice alone and “behavior (GO:0007610)”, “membrane organization (GO:0061024)”, and “prion disease—Mus musculus (house mouse) (mmu05020)” in 12-month-old APP-KI mice alone.

The fold changes in the levels of proteins related to the top five biological pathways and processes were examined to determine their upregulation or downregulation. In 5-month-old APP-KI mice, the top five biological pathways and processes were estimated to be upregulated (median: 1.06–1.37-fold, Fig. 2G). In 12-month-old APP-KI mice, “cellular homeostasis (GO:0019725)” (median: 1.19-fold), “membrane organization (GO:0061024)” (median: 1.17-fold), and “neutrophil degranulation (R-MMU-6798695)” (median: 1.25-fold) were estimated to be upregulated, and “behavior (GO:0007610)” (median: 0.902-fold) and “prion disease—Mus musculus (house mouse) (mmu05020)” (median: 0.901-fold) were estimated to be downregulated (Fig. 2G).

Age-dependent biological pathways and processes were also examined. Among the top 5 biological pathways and processes, “neutrophil degranulation (R-MMU-6798695)” and “cellular homeostasis (GO:0019725)” were identified in 5- and 12-month-old APP-KI mice, and the proteins related to these pathways were upregulated in 12-month-old APP-KI mice compared to those in 5-month-old APP-KI mice (Fig. 2H, I). These results indicate significant changes in biological functions in the brain of APP-KI mice following Aβ accumulation.

### Changes in protein expression in isolated brain capillaries following brain Aβ accumulation with aging

**Fig. 3:**
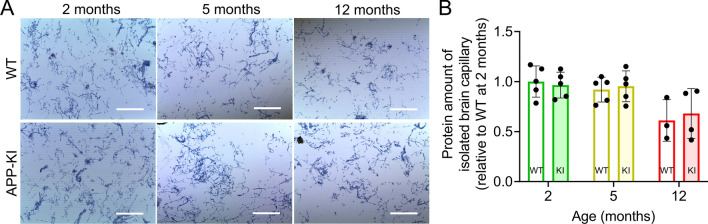
Isolation of brain capillaries of APP-KI mice. **A** Images of isolated brain capillaries from WT and APP-KI mice. The capillaries were stained with trypan blue. Scale bar = 250 µm. **B** Protein levels in isolated brain capillaries of 2-, 5-, and 12-month-old WT and APP-KI mice. The amount of protein was normalized to brain weight. Bars represent the mean ± SD values (n = 3–5). Plotted points represent individual values

To investigate proteomic changes at the BBB during brain Aβ accumulation, capillaries were isolated from the brains of the same 2-, 5-, and 12-month-old APP-KI and WT mice (Fig. 3A). The amount of protein in brain capillaries isolated from APP-KI mice was not significantly different from that in age-matched WT mice (Fig. 3B).

Differentially expressed proteins in the isolated brain capillaries were examined using SWATH-MS-based proteomics. Proteomic data showed low variability in protein intensities and fold changes (%CV < 15%) within each group and age group (Additional file 2: Fig S1C, D), indicating reliable measurements for comparative analysis.

**Fig. 4:**
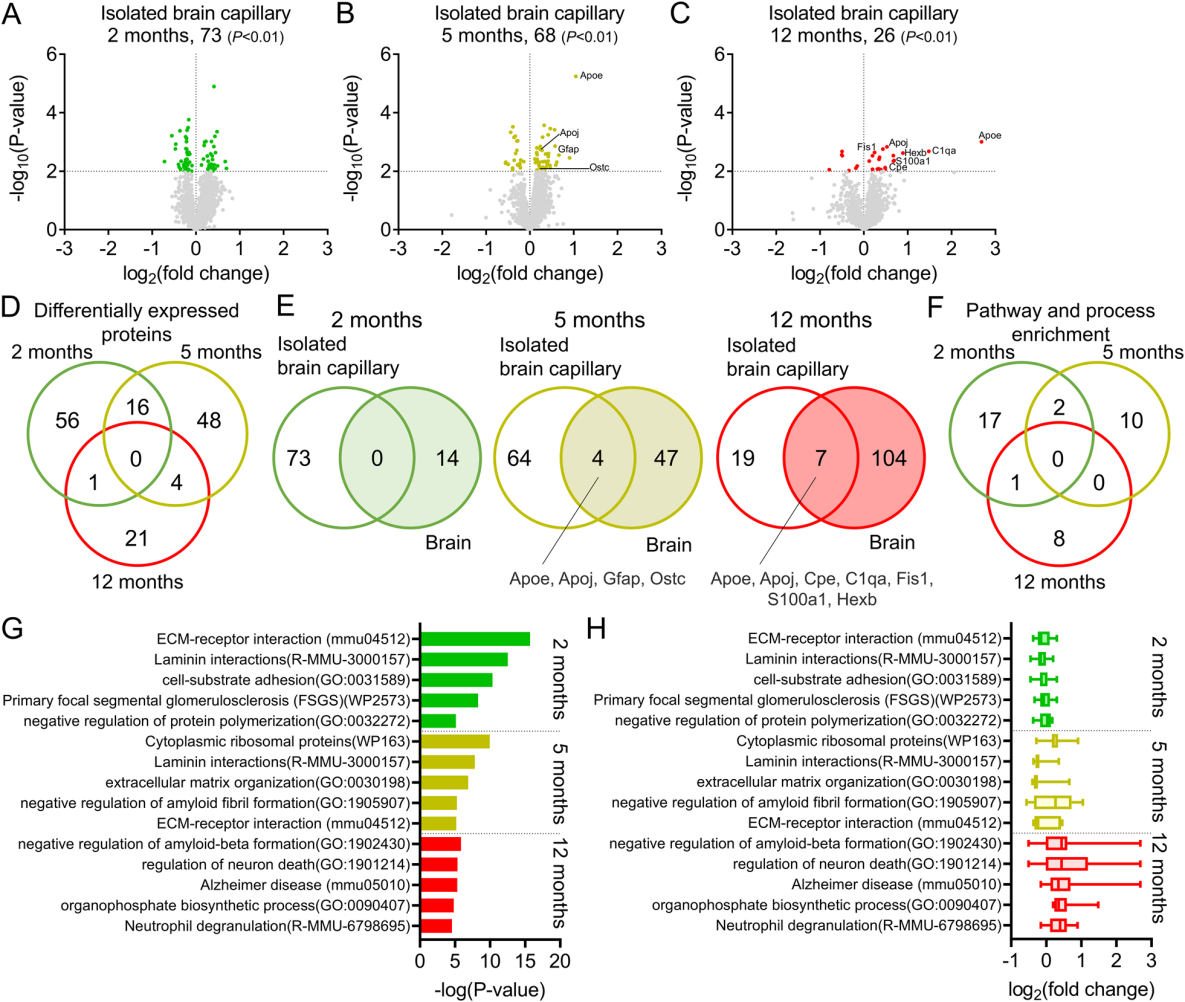
Changes in biological functions based on differentially expressed proteins in the brain capillaries of APP-KI mice. **A**–**C** Volcano plots of the identified proteins in the brain capillaries of 2-, 5-, and 12-month-old mice. Isolated brain capillaries (from 3–5 biological replicates) were subjected to SWATH-MS-based quantitative proteomic analysis. *P*-values estimated using Welch’s t-test were plotted against the fold change (APP-KI/WT) of protein expression for all identified proteins in the isolated brain capillaries from age-matched mice. The horizontal line in each graph represents the level of significance (*P* < 0.01). The vertical line in each graph represents the fold change (1). **D** Venn diagrams comparing differentially expressed proteins in the isolated brain capillaries of 2-, 5-, and 12-month-old APP-KI mice. **E** Venn diagrams comparing differentially expressed proteins between isolated brain capillaries and brains of age-matched APP-KI mice. **F** Venn diagrams comparing the top 20 enriched biological processes identified using Metascape in the isolated brain capillaries of 2-, 5-, and 12-month-old APP-KI mice. **G**, **H** Top 5 enriched biological pathways and processes (**G**) and changes in the associated protein levels (**H**) in 2-, 5-, and 12-month-old APP-KI mice. Box-plot elements: center line, median; box limits, upper and lower quartiles; whiskers, minimum and maximum values

Brain capillary endothelial cell-specific proteins were the most enriched in isolated brain capillaries, but a small number of proteins specifically expressed in astrocytes, microglia, neurons, and oligodendrocytes were also identified (Additional file 2: Fig S2). The numbers of differentially expressed proteins in 2- and 5-month-old APP-KI mice were approximately 2.6-fold higher than those in 12-month-old APP-KI mice (*P* < 0.01, Welch’s t-test; Fig. 4A–C, Additional file 1: Table S2). There were no common differentially expressed proteins among the 2-, 5- and 12-month-old mice (Fig. 4D). To assess whether differentially expressed proteins were selectively altered in isolated brain capillaries, we compared differentially expressed proteins in isolated brain capillaries and brains at the same age in months, and 0 (0%), 4 (5.88%), and 7 (26.9%) of such proteins were identified in both groups (Fig. 4E). Apoe and Apoj were upregulated in an age-dependent manner in both isolated brain capillaries and brains of 5- and 12-month-old APP-KI mice, and this upregulation was well correlated (Additional file 2: Fig S3). The levels of all other proteins were independently altered in brain capillaries and the brain. These results suggest that the changes in protein expression in the brain capillaries and the brain were largely independent.

To identify alterations in the biological functions of the BBB, pathway and process enrichment analyses of differentially expressed proteins were performed using Metascape. The highest number of altered biological pathways and processes were observed in 2-month-old APP-KI mice, and the alterations decreased with age (Fig. 4F). Among the top five altered biological pathways and processes (Fig. 4G), proteins that were enriched in biological pathways and processes related to “ECM-receptor interaction (mmu04512)” and “Laminin interactions (R-MMU-3000157)” were identified in 2- and 5-month-old mice (Fig. 4G), and biological function was estimated to be downregulated (median: 0.903-fold, Fig. 4H). In 5-month-old mice, proteins were enriched in biological pathways and processes related to “Cytoplasmic ribosomal proteins (WP163)”, and this biological function was estimated to be upregulated (median: 1.16-fold). In 5- and 12-month-old mice, proteins were enriched in AD-related biological pathways and processes such as “negative regulation of amyloid fibril formation (GO:1905907)” (median: 1.20-fold), “negative regulation of amyloid-beta formation (GO:1902430)” (median: 1.35-fold), “regulation of neuron death (GO:1,901,214)” (median: 1.35-fold), and “Alzheimer’s disease (mmu05010)” (median: 1.27-fold) (Fig. 4G, H). All these biological functions were upregulated with aging (Fig. 4H). These results suggest that alterations in the functions of the BBB are triggered prior to Aβ accumulation in the brain.

### Impairment of basement membrane in the brain capillaries of APP-KI mice

**Fig. 5:**
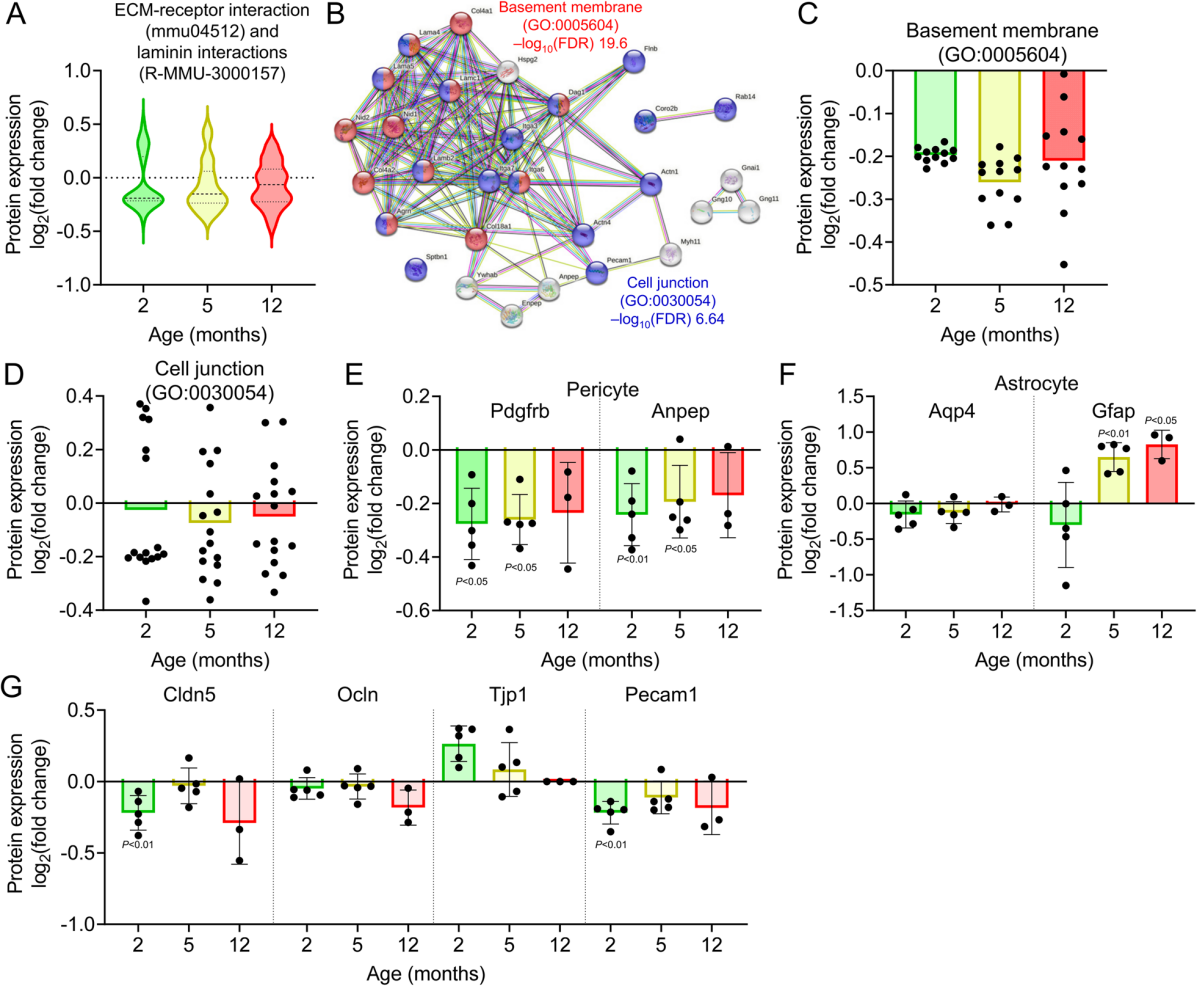
Changes in protein expression related to BBB integrity in isolated brain capillaries of APP-KI mice. **A** Violin plots of protein expression identified in “ECM-receptor interaction (mmu04512)” and “laminin interactions (R-MMU-3000157)” in the isolated brain capillaries of 2-, 5-, and 12-month-old APP-KI mice. Relative fold changes in protein expression (APP-KI/WT) in age-matched mice were calculated. A total of 29 differentially expressed proteins were identified in the brain capillaries of APP-KI mice. Violin plot elements: center line, median; upper and lower lines, upper and lower quartiles. **B** Network analysis of these proteins using STRING and GO analyses. **C**, **D** Age-dependent changes in differentially expressed proteins associated with “basement membrane” (**C**) and “cell junction” (**D**) in APP-KI mice. Plotted points represent individual proteins. **E**–**G** Age-dependent expression of pericyte-selective proteins (**E**), astrocyte-selective proteins (**F**), and tight or adherens junction proteins (**G**) in brain capillaries of APP-KI mice. Relative fold changes in protein expression (APP-KI/WT) in age-matched mice were calculated, and *P*-values between age-matched APP-KI and WT mice were estimated using Welch’s t-test. Bars represent the mean ± SD values (n = 3–5). Plotted points represent individual values

To gain further insights into the changes in biological functions, we conducted a thorough analysis of the quantitative proteomic data. Violin plots revealed downregulation of proteins associated with “ECM-receptor interaction (mmu04512)” and “laminin interactions (R-MMU-3000157)” in 2- to 12-month-old APP-KI mice (Fig. 5A). Network analysis using STRING indicated that proteins related to “basement membrane (GO: 0005604)” exhibited a downregulation of 12.7–16.5% in 2- to 12-month-old APP-KI mice (Fig. 5C), suggesting a compromised basement membrane in APP-KI mice. Proteins associated with “cell junction (GO: 030054)” showed both upregulated and downregulated patterns (Fig. 5D). The downregulated proteins were predicted to be involved in “focal adhesion (mmu04510)”.

Pericytes, which are embedded in the basement membrane of brain capillary endothelial cells, play a crucial role in regulating BBB permeability and integrity. The expression levels of Pdgfrb and Anpep, which are predominantly expressed in pericytes, were lower in 2- and 5-month-old APP-KI mice than in age-matched WT mice (Fig. 5E). Astrocyte endfeet are also attached to the basement membrane; the expression of Aqp4, which is predominantly expressed in astrocytic endfeet, did not change in APP-KI mice (Fig. 5F), whereas that of Gfap, which is predominantly expressed in astrocytes, was increased in 5-month-old APP-KI mice, suggesting astrocyte activation at the BBB (Fig. 5F).

The integrity of the BBB is regulated by the tight and adherens junction proteins of brain capillary endothelial cells. Analysis of tight junction proteins in 2-month-old APP-KI mice revealed a reduction in Cldn5, an increase in Tjp1 (ZO-1), and no change in Ocln (Fig. 5G). Among the adherens junction proteins, Pecam1 expression was reduced (Fig. 5G). These findings indicate that the impairment of BBB integrity in APP-KI mice occurs prior to Aβ accumulation in the brain.

### Increase of ribosomal protein levels in the brain capillaries of APP-KI mice

**Fig. 6:**
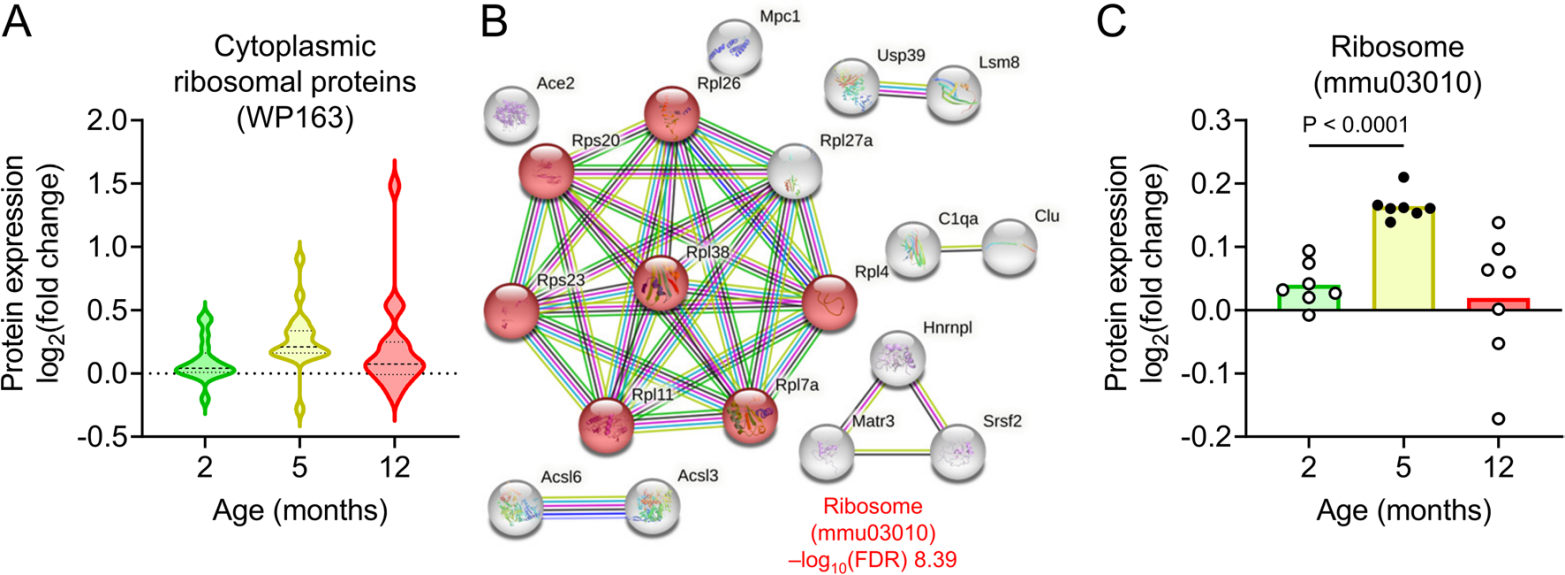
Changes in ribosomal protein expression in isolated brain capillaries of APP-KI mice. **A** Violin plots of protein expressions identified in “Cytoplasmic ribosomal proteins (WP163)” in the isolated brain capillaries of 2-, 5-, and 12-month-old APP-KI mice. Nineteen differentially expressed proteins were identified in the brain capillaries of APP-KI mice. Relative fold changes in protein expression (APP-KI/WT) in age-matched mice were calculated. Violin plot elements: center line, median; upper and lower lines, upper and lower quartiles. **B** Network analysis of the 19 differentially expressed proteins by STRING and GO analysis. **C** Age-dependent changes of differentially expressed proteins associated with “ribosome” in APP-KI mice. Closed circles indicate a significant difference between age-matched WT and APP-KI mice (*P* < 0.01); open circles indicate no significant difference. Bars represent the mean values. Plotted points represent individual proteins. Statistical significance was determined using one-way analysis of variance followed by Tukey’s post hoc test

In the brain capillaries of 5-month-old APP-KI mice, the presence of “Cytoplasmic ribosomal proteins (WP163)” was detected (Fig. 4G). Violin plots also showed that proteins related to “Cytoplasmic ribosomal proteins (WP163)” were upregulated in the brain capillaries of 5-month-old APP-KI mice (Fig. 6A). Through network analysis using STRING and KEGG pathway analysis, we identified seven large subunits of ribosomal proteins (Rpl) and small subunits of ribosomal proteins (Rps) that play crucial roles in maintaining the structure and function of the ribosome (Fig. 6B). The expression of these proteins was increased in the brain capillaries of 5-month-old APP-KI mice compared to that in 2-month-old APP-KI mice (Fig. 6C). Venn diagrams illustrated differential abundance of Rpl and Rps in both brain capillaries and the brain (Additional file 2: Fig S4A). However, no changes were observed in the differentially expressed proteins related to Rpl and Rps in the age-matched brains (Additional file 2: Fig S4B). Analysis of a single RNA-seq database indicated higher mRNA levels of Rpl4, Rpl7a, Rpl38, and Rps20 in endothelial cells than in other brain cell types [30], and RPL4, RPL7A, and RPS20 are reported to be significantly upregulated in AD patients [31]. These findings suggest a selective increase in ribosomal proteins specifically within the brain capillaries during the process of brain Aβ accumulation.

### Effect of increased Apoe and Apoj on alterations in brain function

**Fig. 7:**
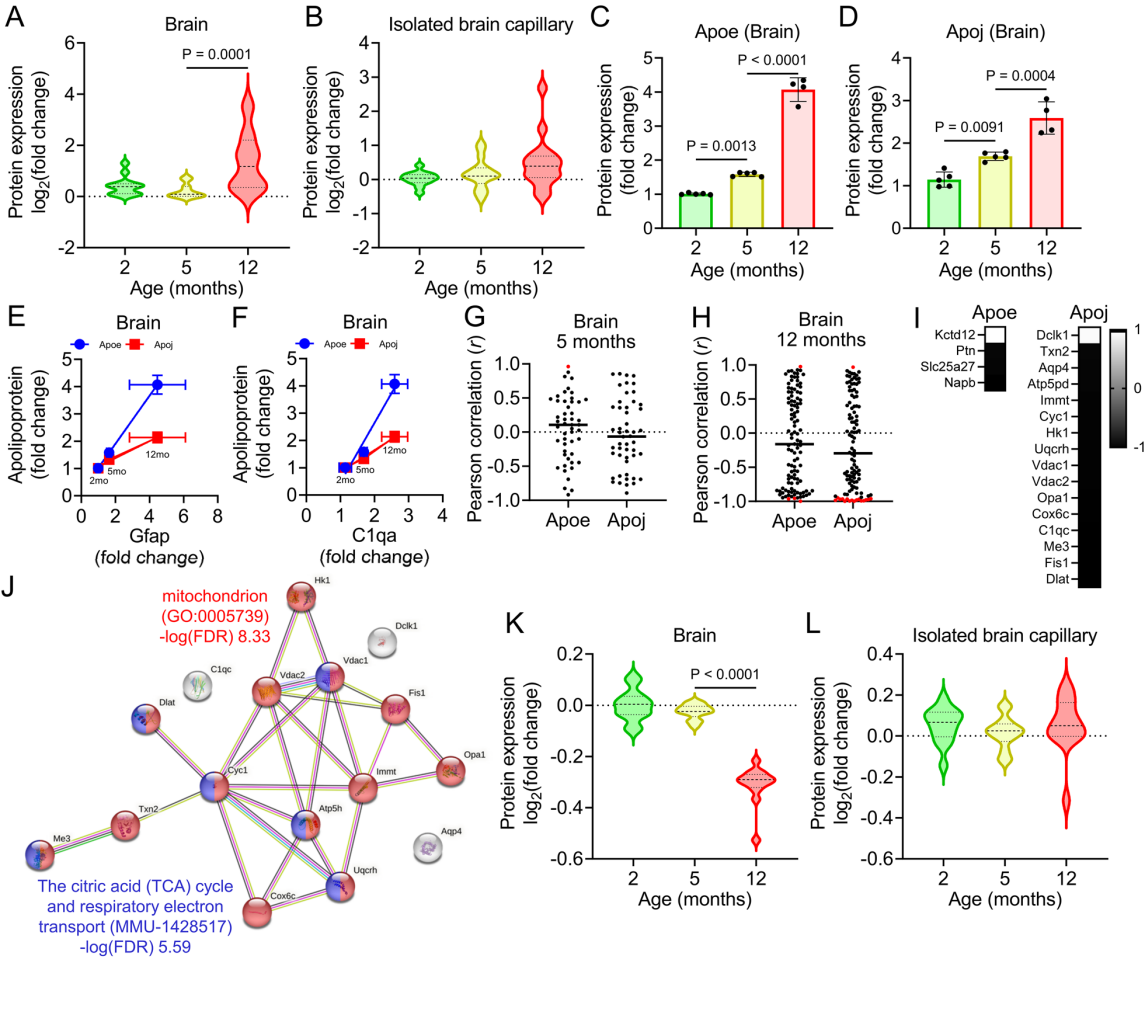
Effects of age-dependent upregulation of Apoe and Apoj on brain function in APP-KI mice. **A**, **B** Violin plots of AD-related protein expression in the brain (**A**) and isolated brain capillaries (**B**) of 2-, 5-, and 12-month-old APP-KI mice. Twenty differentially expressed proteins were identified. Relative fold changes in protein expression (APP-KI/WT) in age-matched mice were calculated. Violin plot elements: center line, median; upper and lower lines, upper and lower quartiles. **C**, **D** Age-dependent changes in Apoe (**C**) and Apoj (**D**) levels in the brains of APP-KI mice. Bars represent the mean ± SD (n = 4–5). Plotted points represent individual values. **E** Correlation between age-dependent changes in Apoe and Gfap or C1qa in the brains of APP-KI mice. **F** Correlation between age-dependent changes in Apoj and Gfap or C1qa in the brains of APP-KI mice. **G**, **H** Correlation analysis of upregulation of Apoe or Apoj with 110 differentially expressed proteins in the brains of 5-month-old (**G**) and 12-month-old (**H**) APP-KI mice. Red circles indicate differentially expressed proteins (*P* < 0.05). **I** Correlation coefficients of differentially expressed proteins with Apoe or Apoj. **J** Network analysis of 16 differentially expressed proteins associated with Apoj expression using STRING and GO analyses. **K**, **L** Age-dependent changes in differentially expressed proteins associated with “mitochondria” in the brain (**K**) and brain capillaries (**L**) of APP-KI mice. Statistical significance was determined using one-way analysis of variance followed by Tukey’s post hoc test. Violin plot elements: center line, median; upper and lower lines, upper and lower quartiles

Network analysis revealed the presence of 20 differentially expressed proteins associated with four AD-related pathways and processes in the brains and brain capillaries of APP-KI mice (Fig. 4G). Violin plots demonstrated that these 20 proteins exhibited increased expression levels in the brains of 12-month-old APP-KI mice (Fig. 7A) and showed a tendency to increase in the capillaries of 12-month-old APP-KI mice (Fig. 7B). Among these proteins, Apoe and Apoj displayed age-dependent upregulation in the brain (Fig. 7C, D). Co-expression analysis suggested a positive correlation between the age-dependent increase in Apoe and Apoj and the expression of Gfap and C1qa, which are known to increase during brain inflammation (Fig. 7E, F). Notably, the age-dependent expression of Gfap and C1qa also exhibited a strong correlation (Additional file 2: Fig S5). These findings indicate that the age-dependent elevation of Apoe and Apoj levels is associated with brain inflammation.

To investigate the potential influence of increased Apoe and Apoj levels on cellular and organelle functions in the brain, correlations between Apoe and Apoj protein expression and differentially expressed proteins (Fig. 2D) were examined in 5- and 12-month-old APP-KI mice. In 5-month-old mice, no co-expressed proteins were detected, except for Fasn and Apoe (Fig. 7G). However, in 12-month-old mice, the Apoe level correlated with that of four proteins (Kctd12, Ptn, Slc25a27, and Napb) involved in neuronal development, secretion, and mitochondria (Fig. 7H, I), whereas the Apoj level correlated with that of 16 proteins (Dclk1, Txn2, Aqp4, Atp5pd, Immt, Cyc1, Hk1, Uqcrh, Vdac1, Vdac2, Opa1, Cox6c, C1qc, Me3, Fis1, and Dlat; Fig. 7I). Further analysis using STRING and GO (Cellular Component and Molecular Function) revealed that six of these 16 proteins were associated with mitochondrial function, such as “The citric acid (TCA) cycle and respiratory electron transport (MMU-1428517)” (Fig. 7J), while 13 proteins were related to “mitochondrion (GO: 0005739)”. Violin plots demonstrated the downregulation of these 13 proteins related to “mitochondrion (GO: 0005739)” in the brain of 12-month-old APP-KI mice (Fig. 7K) but not in the brain capillaries at any age (Fig. 7L). These findings suggest that upregulated Apoe and Apoj levels contribute to impaired brain function in APP-KI mice.

### Immunohistochemical analysis of Apoe and Apoj in 12-month-old APP-KI mice

**Fig. 8:**
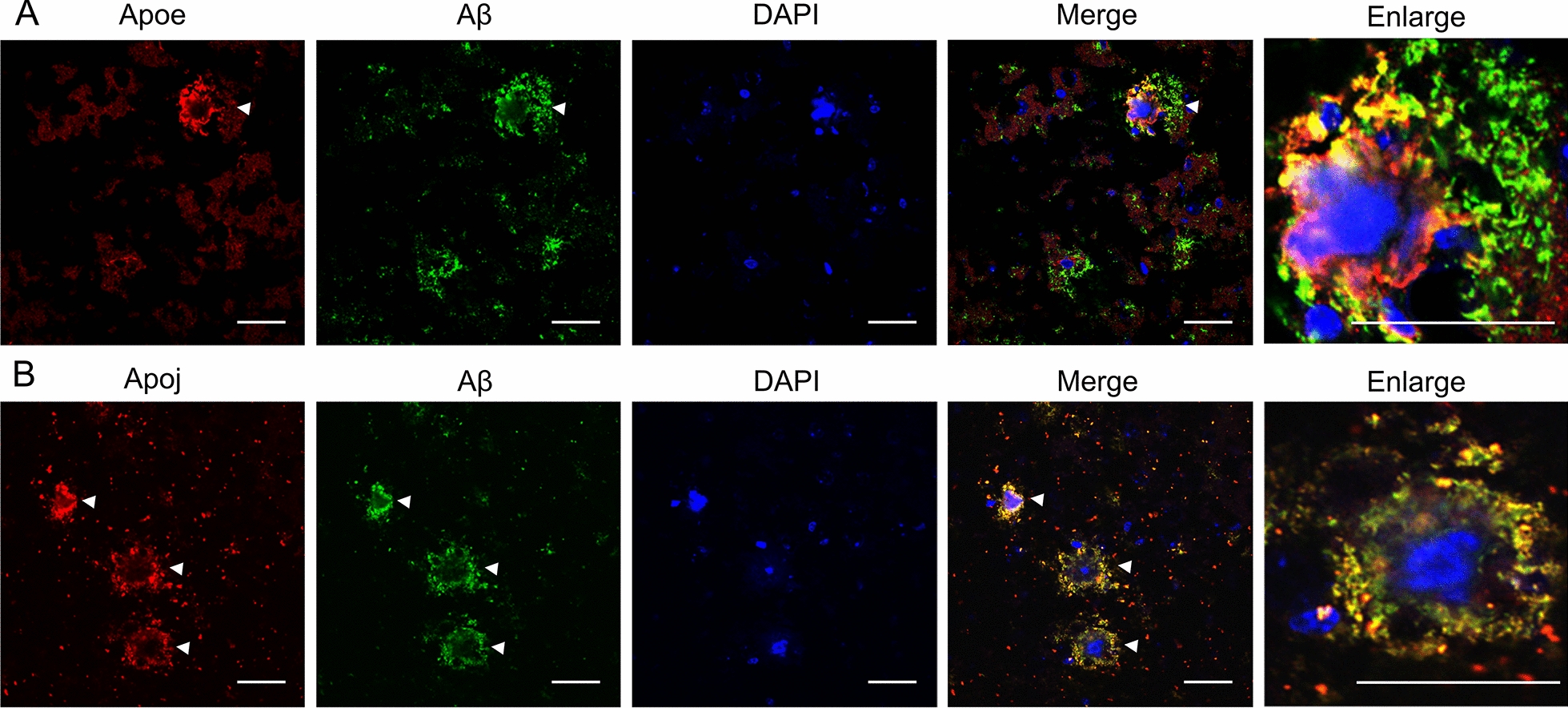
Localization of Apoe and Apoj in amyloid plaques in APP-KI mice. **A** Representative images of colocalization of Apoe and Aβ (82E1) in the cortex of 12-month-old APP-KI mice (n = 3 biological replicates). **B** Representative images of colocalization of Apoj and Aβ in the cortex of 12-month-old APP-KI mice (n = 3 biological replicates). Scale bar = 10 µm

To investigate the accumulation of Apoe and Apoj in the brains of APP-KI mice, immunohistochemical analysis was conducted. In the cortex of 12-month-old APP-KI mice, Apoe was localized within amyloid plaques that were positive for the anti-Aβ antibody (82E1), and there was partial overlap between Apoe and these plaques (Fig. 8A). Similarly, Apoj was detected in amyloid plaques of the cortex in 12-month-old APP-KI mice, with almost all of it localized at the periphery of the plaques (Fig. 8B). These findings indicate that the increased levels of Apoe and Apoj in the brains of APP-KI mice were associated with their binding to amyloid plaques and that Apoe and Apoj occupied distinct regions within the plaques.

### Alterations of transporters in the brain capillaries and brains of APP-KI mice

**Fig. 9:**
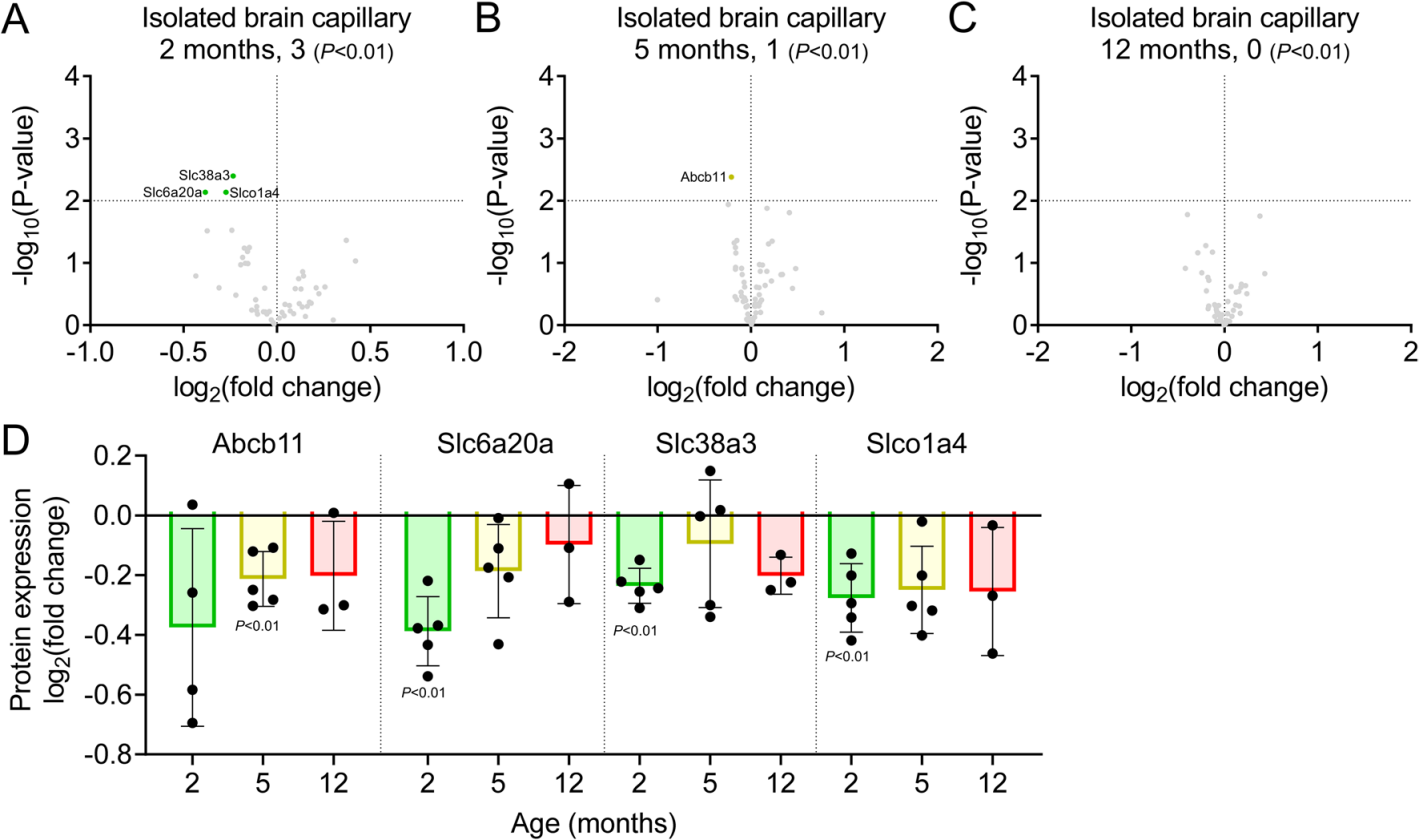
Changes in ABC and SLC transporters in the brain capillaries of APP-KI mice. **A**–**C** Volcano plots of the identified ABC and SLC proteins in the brain capillaries of 2-, 5-, and 12-month-old mice. *P*-values estimated using Welch’s t-test were plotted against the fold change (APP-KI/WT) of protein expression for ABC and SLC transporter proteins in the isolated brain capillaries from age-matched mice. The horizontal line in each graph represents the level of significance (*P* < 0.01). The vertical line in each graph represents the fold change (1). **D** Age-dependent expression of differentially expressed ABC and SLC transporter proteins in the brain capillaries of APP-KI mice. Relative fold changes in protein expression (APP-KI/WT) in age-matched mice were calculated, and *P*-values were estimated using Welch’s t-test. Bars represent the mean ± SD values (n = 4–5). Plotted points represent individual values

We identified nine ABC transporters and 55 SLC transporters in the brain capillaries (Fig. 9A–C), and the age-dependent changes in their expression levels are summarized in Additional file 1: Table S3. Among these, one ABC and three SLC transporters (Abcb11 in 5-month-old mice, *P* < 0.01, Fig. 9D, and Slc6a20a, Slc38a3, and Slco1a4 in 2-month-old mice; *P* < 0.01, Fig. 9D) were differentially expressed. The expression of these proteins was downregulated in 2–12-month-old APP-KI mice. However, the major BBB ABC transporters, such as Abcb1a/P-gp, Abcc4/Mrp4, and Abcg2/Bcrp, and SLC transporters, such as Slc2a1/Glut1, Slc3a2/4F2hc, Slc7a5/Lat1, and Slc16a1/Mct1, showed no alterations (*P* < 0.01, Additional file 1: Table S3).

**Fig. 10:**
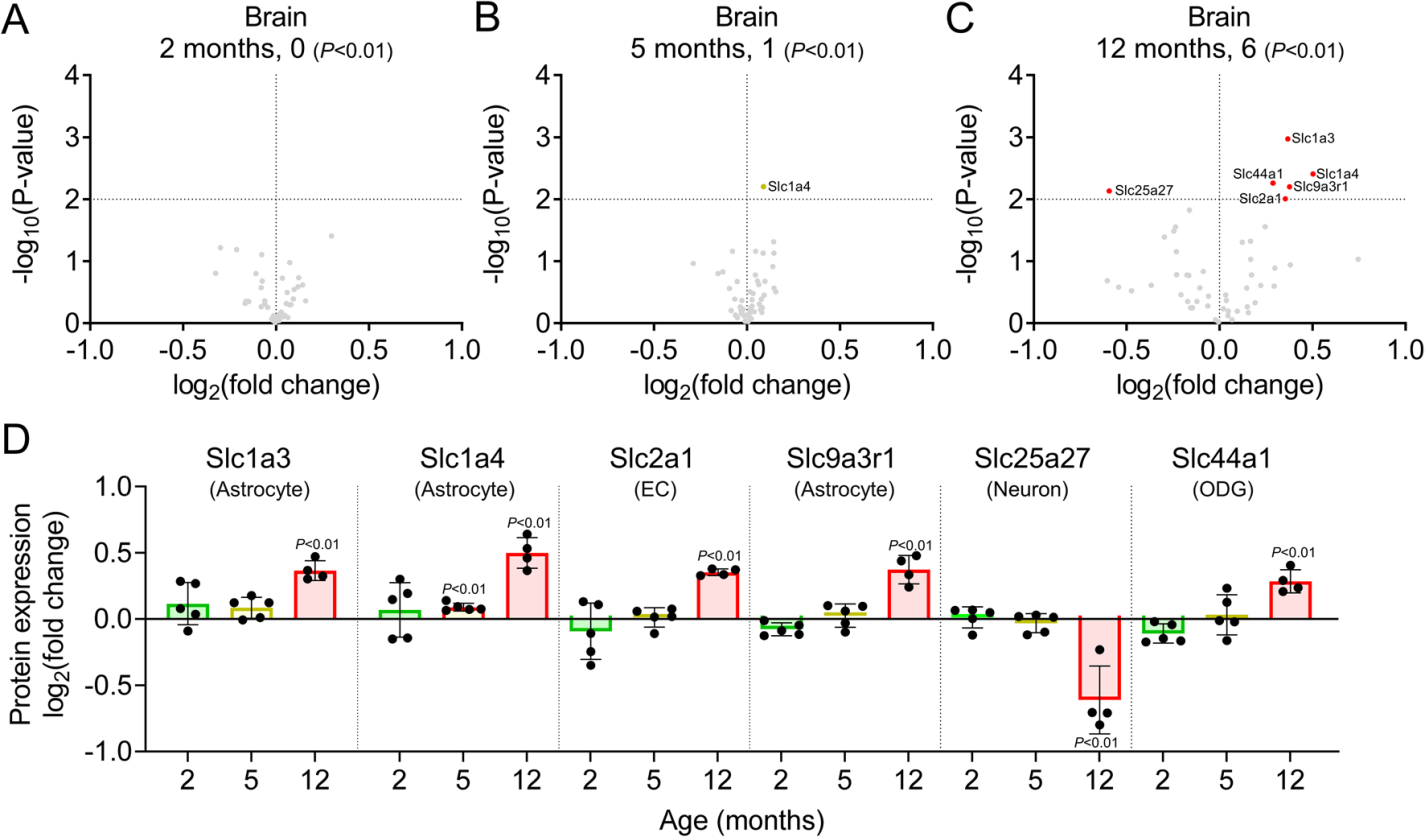
Changes in ABC and SLC transporter levels in APP-KI mouse brain. **A**–**C** Volcano plots of the identified ABC and SLC proteins in the brains of 2-, 5-, and 12-month-old mice. *P*-values estimated by Welch’s t-test were plotted against fold change (APP-KI/WT) of protein expression for ABC and SLC transporter proteins in the brain lysates of age-matched mice. The horizontal line in each graph represents the significance level (*P* < 0.01). The vertical line in each graph represents the fold change (1). **D** Age-dependent expression of differentially expressed ABC and SLC transporter proteins in the brains of APP-KI mice. Relative fold changes in protein expression (APP-KI/WT) in age-matched mice were calculated, and *P*-values were estimated using Welch’s t-test. SLC transporters were classified based on the cells primarily expressing them (endothelial cells [EC], neurons, astrocytes, and oligodendrocytes [ODG]), according to the RNA-seq database (26, 27). Bars represent the mean values (n = 4–5). Plotted points represent individual values

ABC and SLC transporters expressed in brain cells also play significant roles in the maintenance of brain function. We identified 56 SLC transporters in the brain, and the age-dependent changes in their expression are summarized in Additional file 1: Table S4. No differentially expressed ABC transporter proteins were detected in the brains of APP-KI mice at any age in this study (Additional file 1: Table S4). In contrast, the differential abundance of SLC transporters was more pronounced in 12-month-old APP-KI mice than in 2- and 5-month-old mice (Fig. 10A–C). Among the identified SLC transporters, six showed significant differences in the brains of 2-, 5-, and 12-month-old APP-KI mice compared to age-matched WT mice (Slc1a4 in 5- and 12-month-old and Slc1a3, Slc2a1, Slc9a3r1, Slc25a27, and Slc44a1 in 12-month-old mice; *P* < 0.01, Welch’s t-test). According to the RNA expression levels in the RNA-seq database, these six SLC transporters are localized in the endothelium, neurons, astrocytes, microglia, and oligodendrocytes, with at least threefold higher expression compared to that in other brain cells [30, 32]. The level of Slc2a1/Glut1, which is primarily expressed in brain capillary endothelial cells and at a very low level in astrocytes, increased by 1.28-fold in 12-month-old brains (*P* = 0.00985, Fig. 10D), but not in 12-month-old brain capillaries. The level of Slc25a27, which is primarily expressed in neuronal mitochondria, significantly decreased by 33.7% (*P* = 0.00740) in 12-month-old APP-KI mice (Fig. 10D). The levels of SLC amino acid transporters Slc1a3 and Slc1a4 (primarily expressed in astrocytes) and Slc9a3r1 (a Na^+^/H^+^ exchanger regulatory factor) significantly increased by 1.29-fold (*P* = 0.00107), 1.42-fold (*P* = 0.00392), and 1.30-fold (*P* = 0.00630), respectively, in 12-month-old APP-KI mice (Fig. 10D). The level of Slc44a1, expressed in oligodendrocytes, increased in 12-month-old APP-KI mice (*P* = 0.00550, Fig. 10D). These findings suggest that the levels of SLC transporters in the brain are significantly altered after Aβ accumulation, whereas in the BBB, their expression changes before accumulation.

### Alterations in proteins related to receptor-mediated transcytosis

Proteins involved in receptor-mediated transcytosis (RMT), such as transferrin receptor (Tfrc), insulin receptor, low-density lipoprotein receptor-related protein 1 (Lrp1), CD98 (4F2hc), and SLC2A1, play crucial roles in facilitating the transport of biopharmaceuticals across the BBB into the brain [33–37]. The Tfrc level was reduced by 24.1% in 12-month-old APP-KI mice (*P* = 0.0208); however, the expression of other RMT receptors in the brain capillaries of APP-KI mice remained unchanged (Additional file 1: Table S5). Antibody therapy holds great promise as a potential approach for improving the treatment and management of AD. The neonatal Fc receptor is a key protein involved in the efflux of IgG antibodies from the brain across the BBB [38, 39]; however, its expression showed no significant changes in the brain capillaries of APP-KI mice (Additional file 1: Table S5).

### Protein alterations related to Aβ production and clearance in the brain

Aβ is produced from APP via sequential cleavage by β-secretase and γ-secretase in the brain. The APP level in the brains of 12-month-old APP-KI mice was 4.2% lower than that in WT mice; however, the levels of proteins associated with β-secretase and γ-secretase listed in Additional file 1: Table S6 were below the detection limit.

Several proteins are involved in Aβ clearance in the brain, including neprilysin (Mme), angiotensin-converting enzyme (ACE), endothelin-converting enzyme 1 (Ece1) and Ece2, insulin-degrading enzyme (Ide), and Lrp1. Among these, Ide level was significantly increased in 5-month-old APP-KI mice (1.69-fold; *P* < 0.01), whereas Lrp1 level was increased in 12-month-old APP-KI mice (*P* = 0.0119). Ece1 was detected but did not show any alterations in 5-month-old mice. Mme, ACE, and Ece2 were not detected (Additional file 1: Table S6).

In terms of Aβ clearance at the BBB, receptor for advanced glycation end products (RAGE) is involved in the influx of Aβ into the brain across the BBB, while Lrp1, Abcb1, Abcg2, and Ide are involved in the efflux of Aβ from the brain across the BBB [19, 40–44]. Abcg2 expression was reduced in 5-month-old mice (*P* = 0.0437), but the expression of other proteins did not show any reduction at any age (Additional file 1: Table S6). Lrp2 and RAGE were not identified in the brain capillaries of APP-KI mice.

## Discussion

Aβ accumulation in the brain is a key event in AD pathogenesis. In APP-KI mice, this accumulation becomes visible in 2-month-old APP-KI mice and almost saturates the brain in 7–9-month-old mice [22]. The present proteomic study suggests that impairment of BBB integrity occurs before and during Aβ accumulation. Specifically, proteins related to the basement membrane and tight junctions are impaired before Aβ accumulation, reducing BBB integrity. Moreover, BBB protein expression was not significantly altered after Aβ accumulation. APP-KI mice show increased Aβ42 production and a high Aβ42/Aβ40 ratio [22], and the clearance of Aβ42 through the BBB is estimated to be slower than that of Aβ40 [45]. Aβ42 also easily forms dimers and oligomers that are more toxic than Aβ monomers [46, 47]. Soluble Aβ42 levels are similar in 2- and 9-month-old APP-KI mice, while insoluble and amyloid plaques dramatically increase in 4-month-old APP-KI mice [22]. These findings suggest that soluble Aβ molecules, including monomers, dimers, and oligomers, impair BBB integrity prior to Aβ accumulation.

Network analysis suggested that the expression levels of four basement membrane proteins (collagen IV, laminin, nidogen, and perlecan) were reduced at the early, intermediate, as well as advanced stages of Aβ accumulation. This finding is consistent with the reported pathological brain microvascular basement membrane alterations in AD [2, 4]. Collagen IV is required for basement membrane maintenance [48], and loss of COL4A1 in both basement membrane epithelial cells and pericytes contributes to cerebrovascular defects [49, 50]. However, the role of basement membrane changes in AD and the detailed mechanisms involved have not been elucidated, and further studies will be needed to clarify these aspects.

Pericytes are embedded in the basement membrane of brain capillaries and are critical for proper BBB function [51]. Pericyte loss facilitates AD pathology in APP*-*overexpressing Swedish patients with mutations in the human APP gene (*APP*^*sw/0*^) [52]. Pdgfrb expression was reduced in 2-month-old APP-KI mice, similar to the reduction in basement membrane protein expression at the same age. The binding of laminin to pericytes is primarily facilitated by integrins present on the pericyte surface. In this study, the expression of two laminin isoforms predicted to be expressed in pericytes, α4β2γ1 and α5β2γ1, was estimated to be reduced in isolated brain capillaries. Integrins α3, α6, and α7 form complexes with integrin β1, which in turn binds to laminin, and these integrin complexes were also estimated to be expressed in pericytes, suggesting that basement membrane integrity in the brain capillaries is impaired in the early phase of AD progression, possibly causing pericyte loss.

Network analysis also suggested synaptic and mitochondrial impairment during and after Aβ accumulation. Gfap, C1qa, and C1qb levels increased during and after accumulation, suggesting that inflammation was induced during accumulation. Furthermore, the cerebral metabolic rate of glucose is expected to decrease after Aβ accumulation due to the reduced expression of Slc2a3 (which has a major role in neuronal glucose uptake) by 10.6% (*P* = 0.0150, Additional file 1: Table S4) and hexokinase 1 (a key player in glycolysis) by 18.2% (*P* = 0.00908, Additional file 1: Table S1), as well as Slc25a27 (predominantly localized in neuronal mitochondria), in 12-month-old APP-KI mice. Brain insulin signaling is also supposedly reduced during Aβ accumulation, as shown by the increase in Ide expression, which is assumed to facilitate insulin degradation in the brain. Therefore, our present findings suggest that BBB degradation and neuronal damage occur at different times during the Aβ accumulation process, which is probably related to different forms of Aβ. This also aligns with the clinical evidence from patients with AD, which indicates that BBB degradation occurs during early AD progression [7–12].

SLC transporters in the BBB have a significant role in nutrient transport, and their alterations cause neuronal disorders such as GLUT1 deficiency syndrome [53] and cerebral creatine deficiency syndrome [54]. Slc6a20a, Slc38a3, Slc22a8, and Slco1a4 levels were reduced in the BBB of 2-month-old APP-KI mice. Slc6a20a regulates N-methyl D-aspartate receptor (NMDAR) function in the mouse brain by modulating proline and glycine levels [55]. Slc38a3 is a transporter of neutral amino acids such as glutamine [56], and *Slc38a3-*mutant mice are ataxic due to higher brain levels of glutamine and reduced glutamate and gamma-aminobutyric acid levels [57]. Slc22a8 plays an important role in eliminating endogenous and exogenous organic anions and contributes to the brain-to-blood efflux of homovanillic acid, a major toxic dopamine metabolite [58]. A previous study reported elevated homovanillic acid levels in the cerebrospinal fluid (CSF) of patients with AD [59].

SLC transporters in the brain also play a critical role in the regulation of neurotransmitters, and their dysfunction in the BBB is also a cause of neurodegenerative diseases [60]. Slc1a4 (ASCT1), which is predominantly expressed in astrocytes, was upregulated in the brains of 12-month-old APP-KI mice. Slc1a4 modulates D-serine levels in the brain and regulates synaptic plasticity and behavior by binding to NMDARs. D-serine was found to be significantly upregulated in the hippocampus and parietal lobes of patients with AD post-mortem and in the CSF of probable AD patients compared to subjects without dementia [61]. Thus, Slc1a4 reduction may contribute to an increase in D-serine levels in the brain interstitial fluid. Slc1a3, predominantly expressed in astrocytes, is responsible for glutamine uptake in the brain [62]. Furthermore, brain glucose transport may be affected by alterations in SLC2A1/GLUT1 and SLC2A3/GLUT3 levels. Neuronal overexpression of GLUT1 in a *Drosophila* model of AD alleviated neurodegeneration and behavioral alterations and counteracted Aβ toxicity [63]. Thus, alterations in brain homeostasis due to changes in SLC transporter expression may be involved in the progression of AD.

ABCB1/P-gp, ABCC4/MRP4, and ABCG2/BCRP are key efflux transporters in the BBB. Immunohistochemistry and PET studies have shown reduced P-gp expression and activity in patients with AD [16–18, 64, 65]; however, P-gp expression in the brain capillaries was stable in APP-KI mice in our study. Insulin resistance in patients with diabetes increases the risk of AD [66]. The insulin receptor is expressed in brain capillary endothelial cells [67], and we previously reported that insulin signaling in human BBB-model cells in vitro regulates P-gp expression [68], cell proliferation, and tight-junction integrity [69]. P-gp expression is downregulated in streptozotocin-treated rats with type 1 [70, 71] and type 2 diabetes [72]. Additionally, protein expression and function of P-gp were reduced in mice on a high-fat diet [23]. Thus, we assumed that the decreased expression of P-gp in the AD brain is caused by the suppression of insulin signaling in the BBB.

With regard to aberrant cholesterol metabolism, a systems biology approach identified alterations in cholesterol and bile acid metabolism in AD, and some bile acids detected in the brain may have been transported from the blood [73]. Notably, our results showed that Slco1a4 and Abcb11 levels were reduced before and during Aβ accumulation in APP-KI mice. Slco1a4 is a taurocholate transporter in the mouse BBB [74], and Abcb11 is an efflux transporter for bile salts; thus, alterations in these BBB transporters may alter bile acid levels in the AD brain.

ApoE, a major lipid transport vehicle in the brain, is the most common genetic risk factor for AD. ApoE is associated with the formation of amyloid plaques [75], and ApoE immunotherapy is effective in ameliorating amyloid pathology because it targets ApoE in the plaque core and cerebral blood [76]. We found that Apoe levels increased in the APP-KI mouse brain during and after Aβ accumulation and in the BBB of 12-month-old APP-KI mice, and that Apoe was abundant in the core of amyloid plaques in 12-month-old mice. Apoe is secreted by astrocytes; moreover, non-lipidated Apoe is prone to aggregation, whereas the lipidated form reduces aggregation [77]. Apoe is lipidated principally through the action of Abca1 [78, 79], and Abca1 and Apoe expression was increased in 12-month-old APP-KI mice (the expression of other RMT receptors was not altered in the brain capillaries of APP-KI mice). However, the increase in Abca1 was only 1.4-fold in these mice (Additional file 2: Fig S6), whereas Apoe increased by fourfold. These findings suggest that non-lipidated ApoE levels increase during and after Aβ accumulation, accelerating amyloid plaque deposition.

ApoJ, secreted predominantly by astrocytes, is the third most common genetic risk factor for AD, although its role in the pathogenic mechanisms remains unclear. We found that a decrease in some mitochondrial proteins was inversely associated with an increase in Apoj in 12-month-old mice. Recently, the non-glycosylated 45-kDa ApoJ isoform was found to be localized in the neuronal mitochondrial matrix, and ApoJ is believed to cause mitochondrial dysfunction in neurons [80]. Moreover, mitochondria regulate glucose metabolism, and several FDG PET studies have revealed abnormalities in cerebral glucose metabolism [81, 82]. Overall, our results suggest that ApoJ affects glucose metabolism in the AD brain, although further studies are necessary to elucidate the underlying molecular mechanisms.

Several candidate AD drugs have been rejected during clinical trials because of their poor distribution across the BBB. Our study suggests that the permeability of small-molecule drugs in APP-KI mice is probably similar to that in WT mice, because the expression levels of major ABC and SLC transporters were not significantly different and the fold changes of proteins with significant differences were small. However, the level of Tfrc, an RMT receptor targeted for efficient biotherapeutic delivery across the BBB [33], was significantly reduced by 20% in the brain capillaries of 12-month-old mice. This finding raises concerns about the distribution and efficacy of anti-TFRC antibody-conjugated biomedicines in crossing the BBB in APP-KI mice.

One limitation of this study was that we only examined proteomic changes in male APP-KI mice. The prevalence and severity of AD are greater in women than in men, and brain amyloid accumulation in female APP-KI mice is greater than that in age-matched male APP-KI mice [83]. Thus, there may be sex-related differences in the changes in biological functions due to changes in protein expression in response to Aβ accumulation.

## Conclusions

In conclusion, our results indicate that BBB integrity appears to be altered even before Aβ accumulation in the brain, whereas brain changes occur afterwards. This finding may help to elucidate AD pathophysiology and predict AD drug distribution across the BBB.

### Supplementary Information

**Additional file 1: Table S1.** Temporal profiles of the levels of differentially expressed proteins in the brain of APP-KI mice. Fold change was calculated using the intensities in the brains of age-matched APP-KI and WT mice as estimated by DIA-NN. P-values were estimated using Welch’s test. Data points represent mean ± SD values (n = 3–5). **Table S2.** Temporal profiles of the levels of differentially expressed proteins in isolated brain capillaries of APP-KI mice. Fold change was calculated using the counts in the isolated brain capillaries of age-matched APP-KI and WT mice as estimated by DIA-NN. P-values were estimated using Welch’s test. Data points represent mean ± SD values (n = 3–5). **Table S3.** Temporal profiles of the levels of significantly different ABC and SLC transporter proteins in the brain capillaries of APP-KI mice. Fold change was calculated using the counts in isolated brain capillaries of age-matched APP-KI and WT mice as estimated by DIA-NN. P-values were estimated using Welch’s test. Data points represent mean ± SD values (n = 3–5). **Table S4.** Temporal profiles of the levels of ABC and SLC transporter proteins in the brain of APP-KI mice. Fold change was calculated using the counts in the brains of age-matched APP-KI and WT mice as estimated by DIA-NN. P-values were estimated using Welch’s test. Data points represent mean ± SD values (n = 3–5). **Table S5.** Temporal profiles of the levels of proteins related to receptor-mediated transcytosis in the brain capillaries of APP-KI mice. Fold change was calculated using the counts in the brains of age-matched APP-KI and WT mice as estimated by DIA-NN. P-values were estimated using Welch’s test. Data points represent mean ± SD values (n = 3–5). **Table S6.** Temporal profiles of Aβ production and clearance in the brain and brain capillaries of APP-KI mice. Fold change was calculated using the counts in the brain and isolated brain capillaries of age-matched APP-KI and WT mice as estimated by DIA-NN. P-values were estimated using Welch’s test. Data points represent mean ± SD values (n = 3–5).

**Additional file 2: Figure S1.** Distribution of the CVs of proteomic data. (A-B) Distribution of %CV values of the brain. The %CV was calculated using the intensities estimated by DIA-NN (A) and fold changes of APP-KI to WT (B) for proteins identified in 3–5 replicates. (C-D) Distribution of %CV values of brain capillaries. The %CV was calculated using the intensities estimated by DIA-NN (C) and fold changes of APP-KI to WT (D) for proteins identified in 3–5 replicates. **Figure S2.** Number of cell-specific proteins quantified by proteomic analysis. Proteins selectively expressed in each cell type were extracted and selected according to the criteria that the mRNA expression ratio (fragments per kilobase of exon per million mapped fragments [FPKM] value in most highly expressed cell types/FPKM value in the second highly expressed cell types) was over 10-fold [30]. **Figure S3.** Correlation of age-dependent changes in Apoe and Apoj in brain capillaries and brains of APP-KI mice. Fold change was estimated using protein levels in the brain of age-matched APP-KI and WT mice. Data points represent mean ± SD values (n = 3–5). **Figure S4.** Changes in ribosomal proteins in the brains of APP-KI mice. (A) Venn diagrams comparing the ribosomal proteins identified in the brains and capillaries of 2-, 5-, and 12-month-old APP-KI mice. (B) Fold changes in seven ribosomal proteins identified as differentially expressed proteins in the brain capillaries of APP-KI mice. The fold change was estimated using protein levels in the brains of age-matched APP-KI and WT mice. Data points represent mean ± SD values (n = 3–5). **Figure S5.** Correlation of age-dependent changes in Gfap and C1qa levels in the brains of APP-KI mice. Fold change was estimated using protein levels in the brains of age-matched APP-KI and WT mice. Data points represent mean ± SD values (n = 3–5). **Figure S6.** Alterations in Abca1 levels in isolated brain capillaries. Fold change was estimated using protein expression in the isolated brain capillaries of age-matched APP-KI and WT mice. P-values were estimated using Welch’s t-test. Data points represent mean ± SD values (n = 3–5).

## Abbreviations

Aβ: Amyloid-β peptide
ABC: ATP-binding cassette
ACE: Angiotensin-converting enzyme
AD: Alzheimer’s disease
APP: Amyloid-β precursor protein
BBB: Blood–brain barrier
CNS: Central nervous system
CV: Coefficient of variance
CSF: Cerebrospinal fluid
ECE: Endothelin-converting enzyme
Ide: Insulin-degrading enzyme
NMDAR: N-methyl D-aspartate receptor
PBS: Phosphate-buffered saline
PET: Positron emission tomography
RAGE: Receptor for advanced glycation end
RMT: Receptor-mediated transcytosis
SD: Standard deviation
SLC: Solute carrier
Tfrc: Transferrin receptor
WT: Wild-type
