# Supplementary material for: Proteomic alterations in the brain and blood–brain barrier during brain Aβ accumulation in an APP knock-in mouse model of Alzheimer’s disease
Source: Fluids Barriers CNS. 2023 Sep 14;20:66. doi: 10.1186/s12987-023-00466-9 (PMC10500766; doi:10.1186/s12987-023-00466-9)
Supplement: Supplementary file 2 — Additional file 2: Figure S1. Distribution of the CVs of proteomic data. (A-B) Distribution of %CV values of the brain. The %CV was calculated using the intensities estimated by DIA-NN (A) and fold changes of APP-KI to WT (B) for proteins identified in 3–5 replicates. (C-D) Distribution of %CV values of brain capillaries. The %CV was calculated using the intensities estimated by DIA-NN (C) and fold changes of APP-KI to WT (D) for proteins identified in 3–5 replicates. Figure S2. Number of cell-specific proteins quantified by proteomic analysis. Proteins selectively expressed in each cell type were extracted and selected according to the criteria that the mRNA expression ratio (fragments per kilobase of exon per million mapped fragments [FPKM] value in most highly expressed cell types/FPKM value in the second highly expressed cell types) was over 10-fold [30]. Figure S3. Correlation of age-dependent changes in Apoe and Apoj in brain capillaries and brains of APP-KI mice. Fold change was estimated using protein levels in the brain of age-matched APP-KI and WT mice. Data points represent mean ± SD values (n = 3–5). Figure S4. Changes in ribosomal proteins in the brains of APP-KI mice. (A) Venn diagrams comparing the ribosomal proteins identified in the brains and capillaries of 2-, 5-, and 12-month-old APP-KI mice. (B) Fold changes in seven ribosomal proteins identified as differentially expressed proteins in the brain capillaries of APP-KI mice. The fold change was estimated using protein levels in the brains of age-matched APP-KI and WT mice. Data points represent mean ± SD values (n = 3–5). Figure S5. Correlation of age-dependent changes in Gfap and C1qa levels in the brains of APP-KI mice. Fold change was estimated using protein levels in the brains of age-matched APP-KI and WT mice. Data points represent mean ± SD values (n = 3–5). Figure S6. Alterations in Abca1 levels in isolated brain capillaries. Fold change was estimated using protein expression in the isolat [file 12987_2023_466_MOESM2_ESM.pdf]

Supplemental Information

Proteomic alterations in the brain parenchyma and blood-brain barrier during brain Aβ accumulation in an APP knock-in mouse model of Alzheimer's disease

Shingo Ito[1,2*], Ryotaro Yagi[2], Seiryo Ogata[2,], Takeshi Masuda[1,2], Takashi Saito[3], Takaomi Saido[4], Sumio Ohtsuki[1,2]

[1]Department of Pharmaceutical Microbiology, Faculty of Life Sciences, Kumamoto University, 5–1 Oe-honmachi, Chuo-ku, Kumamoto 862–0973, Japan

[2]Department of Pharmaceutical Microbiology, Graduate School of Pharmaceutical Sciences, Kumamoto University, 5–1 Oe-honmachi, Chuo-ku, Kumamoto 862–0973, Japan.

[3]Department of Environmental Medicine and Molecular Toxicology, Tohoku University Graduate School of Medicine, 2-1 Seiryo-machi, Aoba-ku, Sendai 980-8575, Japan.

[4]Department of Neurocognitive Science, Institute of Brain Science, Nagoya City University Graduate School of Medical Sciences, Kawasumi, Mizuho-cho, Mizuho-ku Nagoya 467–8601, Japan.

[5]RIKEN Center for Brain Science, Laboratory for Proteolytic Neuroscience, 2–1 Hirosawa, Wako, Saitama 351–0198 Japan.

*Corresponding author: Shingo Ito, PhD

Department of Pharmaceutical Microbiology, Faculty of Life Sciences, Kumamoto University, 5–1 Oe-honmachi, Chuo-ku, Kumamoto 862–0973, Japan

Tel: +81-96-371-4327

Fax: +81-96-371-4329

E-mail: ishingo@kumamoto-u.ac.jp

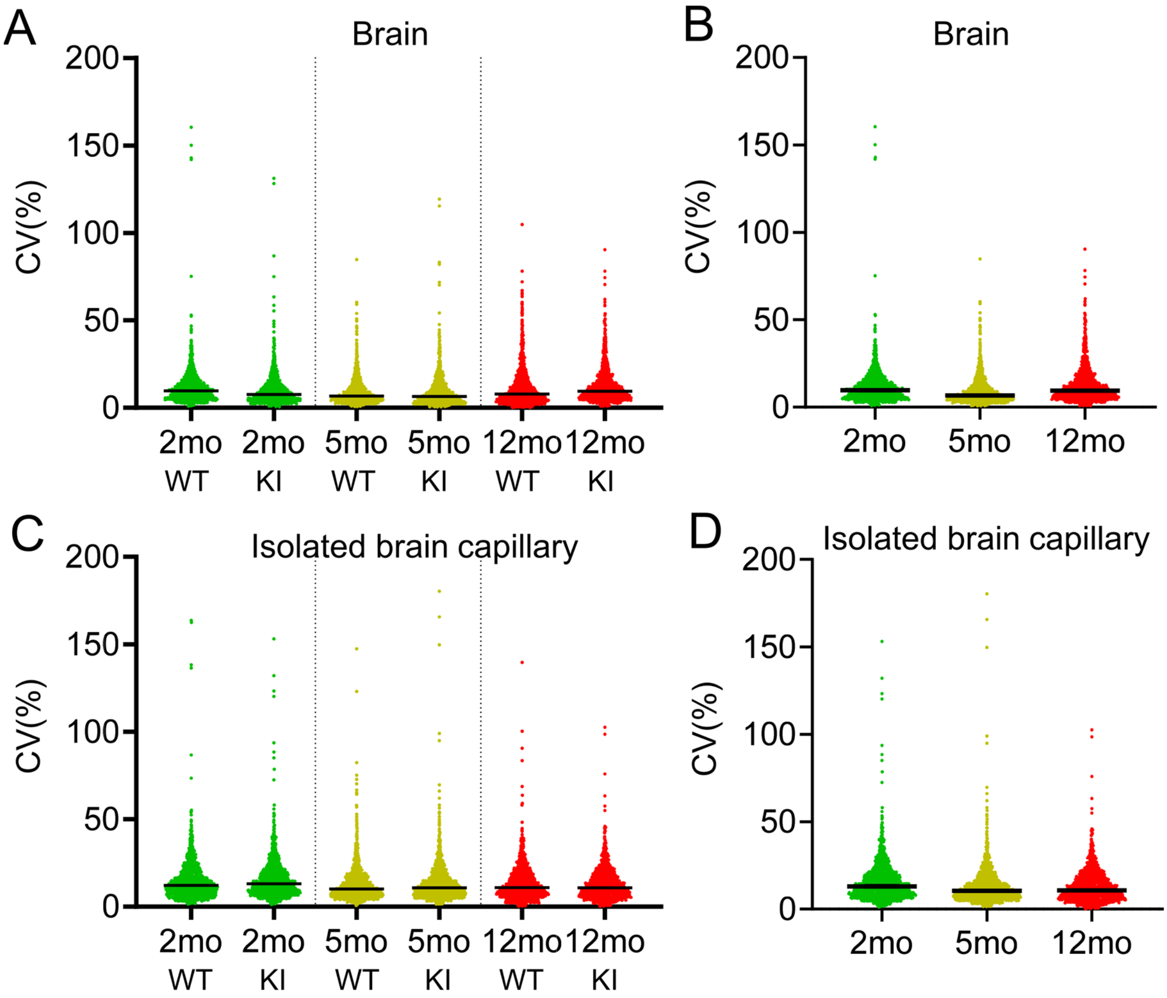

Additional Figure 1. Distribution of the CVs of proteomic data

(A-B) Distribution of %CV values of the brain. The %CV was calculated using the intensities estimated by DIA-NN (A) and fold-changes of APP-KI (KI) to wild type (WT) mice (B) for proteins identified in 3–5 replicates. (C-D) Distribution of %CV values of brain capillaries. The %CV was calculated using the intensities estimated by DIA-NN (C) and fold-changes of APP-KI to WT mice (D) for proteins identified in 3–5 replicates.

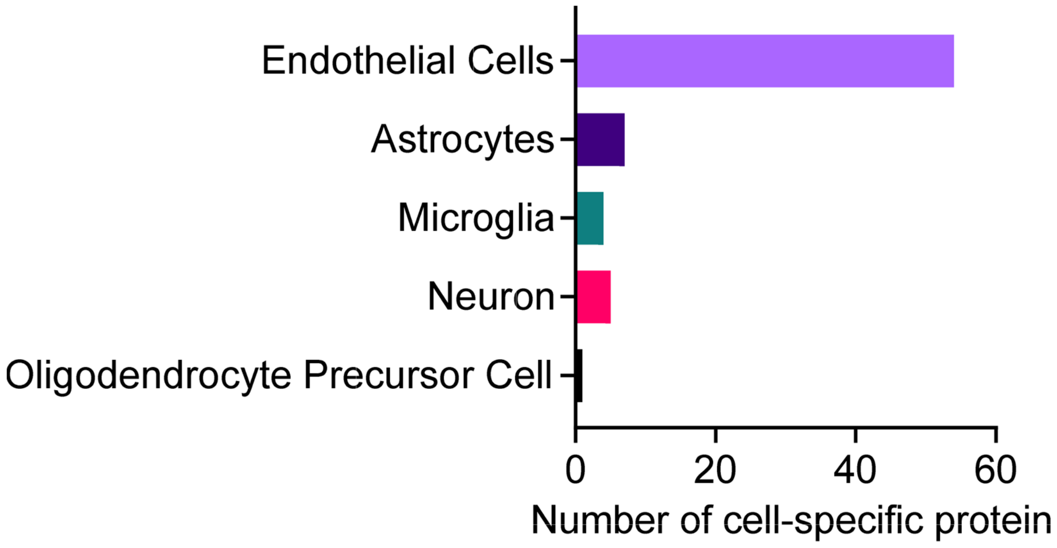

Additional Figure 2. Number of cell-specific proteins quantified by proteomic analysis

Proteins selectively expressed in each cell type were extracted and selected according to the criteria that the mRNA expression ratio (fragments per kilobase of exon per million mapped fragments [FPKM] value in most highly expressed cell types/FPKM value in the second highly expressed cell types) was over 10-fold (30).

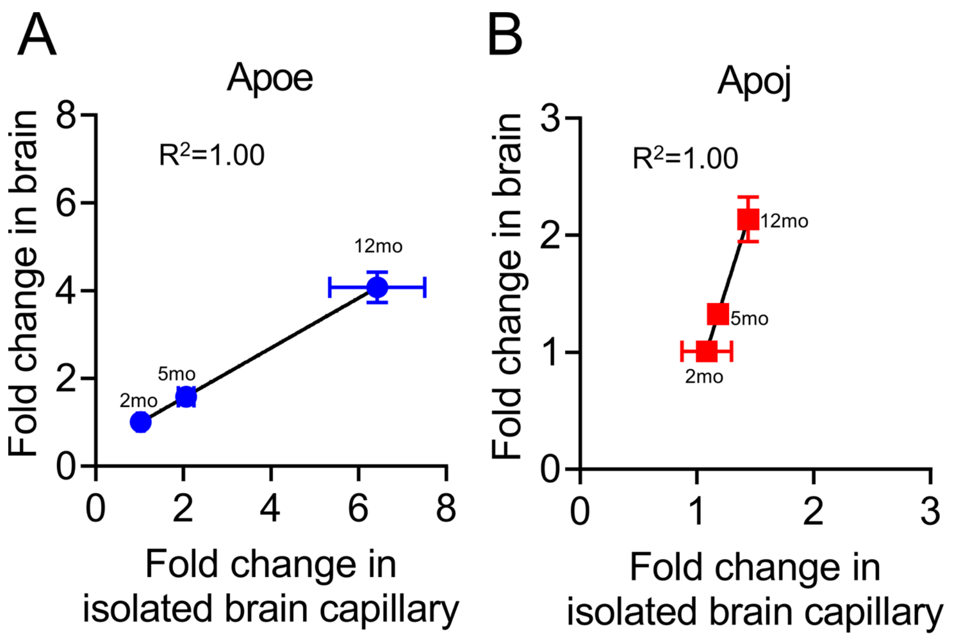

Additional Figure 3. Correlation of age-dependent changes in Apoe and Apoj in brain capillaries and brains of APP-KI mice

Fold-change was estimated using protein levels in the brain of age-matched APP-KI and WT mice. Data points represent mean ± SD values (n = 3–5).

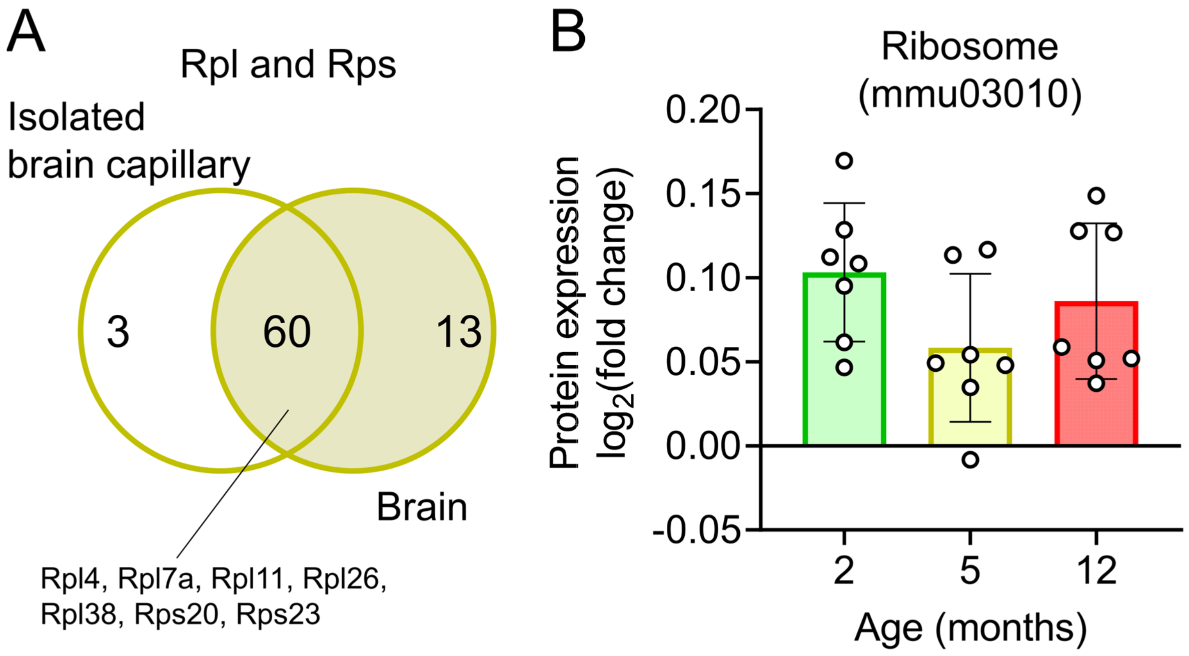

Additional Figure 4. Changes in ribosomal proteins in the brains of APP-KI mice

(A) Venn diagrams comparing the ribosomal proteins identified in the brains and capillaries of 2-, 5-, and 12-month-old APP-KI mice. (B) Fold-changes in seven ribosomal proteins identified as differentially expressed proteins in the brain capillaries of APP-KI mice. The fold-change was estimated using protein levels in the brains of age-matched APP-KI and WT mice. Data points represent mean ± SD values (n = 3–5).

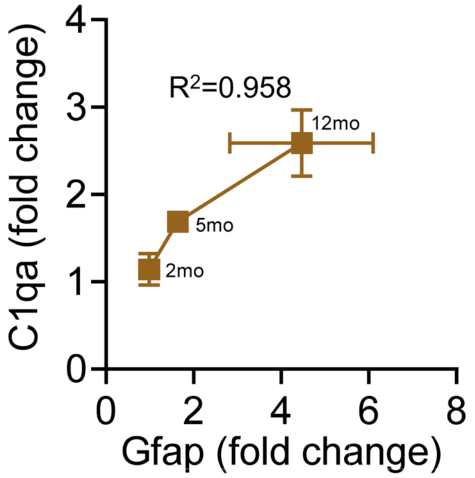

Additional Figure 5. Correlation of age-dependent changes in Gfap and C1qa levels in the brains of APP-KI mice

Fold-change was estimated using protein levels in the brains of age-matched APP-KI and WT mice. Data points represent mean ± SD values (n = 3–5).

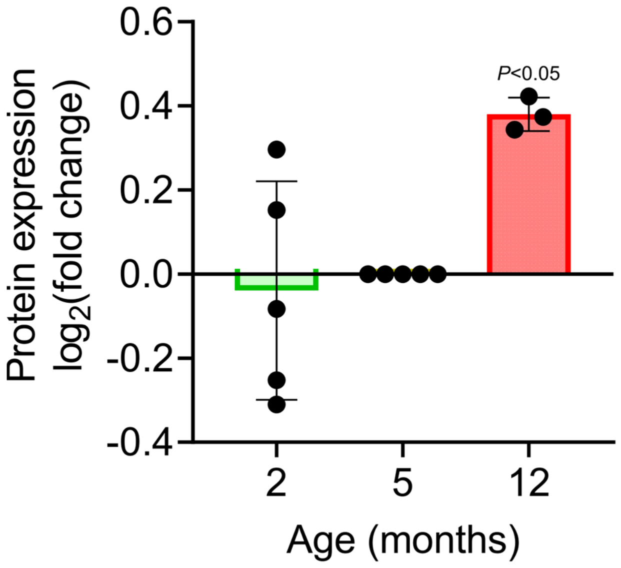

Additional Figure 6. Alterations in Abca1 levels in isolated brain capillaries

Fold-change was estimated using protein expression in the isolated brain capillaries of age-matched APP-KI and WT mice. *P*-values were estimated using Welch's t-test. Data points represent mean ± SD values (n = 3–5).
